# Unusual DNA-binding properties of the *Arabidopsis thaliana* WRKY50 transcription factor at target gene promoters

**DOI:** 10.1007/s00299-020-02611-2

**Published:** 2020-10-02

**Authors:** Konstantin Kanofsky, Jendrik Rusche, Lea Eilert, Fabian Machens, Reinhard Hehl

**Affiliations:** 1 Institut für Genetik, Technische Universität Braunschweig, Spielmannstr. 7, 38106 Braunschweig, Germany; 2 Present Address: Max-Planck-Institut für Molekulare Pflanzenphysiologie, Potsdam Science Park, Am Mühlenberg 1, Golm, 14476 Potsdam, Germany

**Keywords:** Electrophoretic mobility shift assay, Parsley protoplasts, Pep25, Reporter gene, Transcriptional regulation, Transient gene expression

## Abstract

**Key message:**

**WRKY50 from A. thaliana requires WT-boxes at target gene promoters for activation and binding**.

**Abstract:**

Based on the genome-wide prediction of WRKY50 target genes and the similarity of a WRKY50 binding site to WT-boxes in microbe-associated molecular pattern (MAMP)-responsive *cis*-regulatory modules (CRM), four WT-box containing CRMs from the promoter region of three WRKY50 target genes were investigated for their interaction with WRKY50. These target genes are *DJ1E*, *WRKY30* and *ATBBE4.* Two of the four CRMs, one from *DJ1E* and one from *WRKY30*, were able to activate reporter gene expression in the presence of WRKY50. Activation requires the WT-boxes GGACTTTT, GGACTTTG from *DJ1E* and GGACTTTC from *WRKY30*. WRKY50 does not activate a second CRM from *WRKY30* and the CRM from *ATBBE4*, both containing the WT-box TGACTTTT. In vitro gel-shift assays demonstrate WT-box-specific binding of the WRKY50 DNA-binding domain to all four CRMs. This work shows a high flexibility of WRKY50 binding site recognition beyond the classic W-box TTGACC/T.

## Introduction

The transcriptional response of plants towards pathogens or MAMPs is mostly carried out by WRKY transcription factors (TFs). The classic WRKY binding site, the W-box, is enriched in promoters of pathogen-responsive genes (Maleck et al. 2000; Rinerson et al. 2015; Chen et al. 2019). In addition to WRKY TFs, other transcription factor families such as AP2/EREBP, bZIP, and MYB also contribute to pathogen-responsive gene expression (Rushton and Somssich 1998; Amorim et al. 2017). All TFs recognize a characteristic DNA binding site motif and the presence of such sequence motifs in a gene promoter allows the prediction of how the gene is regulated (Hehl and Wingender 2001). To detect such sequences, promoters or gene identification numbers can be submitted to search databases of known regulatory sequences such as TRANSFAC, AGRIS, PlantCare, AthaMap, and PLACE (Higo et al. 1999; Lescot et al. 2002; Matys et al. 2003; Yilmaz et al. 2011; Hehl et al. 2016; Hehl and Bülow 2014). To identify gene groups that may be regulated by the same TF, promoters of co-regulated gene sets can be analysed simultaneously (Galuschka et al. 2007; Hehl and Bülow 2008, 2014). To date, binding sites for almost 1000 of the approximately 2400 TFs of *A. thaliana* have been identified by using mainly high-throughput methods such as DNA Affinity Purification sequencing (DAP-seq) or Protein Binding Microarrays (Perez-Rodriguez et al. 2010; Weirauch et al. 2014; Franco-Zorrilla et al. 2014; O’Malley et al. 2016; Bartlett et al. 2017; Hehl 2017).

In order to discover novel TF-binding sites, pattern recognition programs can be employed, that identify identical sequence patterns in the promoters of co-regulated genes. Examples of such programs include MEME, AlignACE, BioProspector, or CONSENSUS (Bailey and Elkan 1995; Hertz and Stormo 1999; Hughes et al. 2000; Liu et al. 2001). In an earlier study, we applied this approach in combination with transient parsley protoplast transformation technology to identify novel MAMP-responsive sequences (Koschmann et al. 2012). This analysis was based on microarray experiment data obtained from the PathoPlant database. Gene groups induced by up to five different MAMPs were identified and the respective promoter regions were analysed by using the binding-site estimation suite of tools (BEST) (Che et al. 2005; Bülow et al. 2007; Koschmann et al. 2012). Motifs generated from these sequences were then classified using the STAMP webserver (Mahony and Benos 2007). Similarities to binding sites of known plant transcription factor families were determined using the AGRIS, PLACE, and AthaMap databases in STAMP (Higo et al. 1999; Davuluri et al. 2003; Steffens et al. 2004). One of the identified motifs bears similarity to WRKY transcription factor (TF) binding sites (Koschmann et al. 2012). The sequences in this motif are characterized by a conserved GACTTT core sequence. The similarity to W-boxes and the prolonged T-stretch led to the designation WT-box which is an 8-bp sequence harbouring the 6-bp core sequence GACTTT (Machens et al. 2014; Kanofsky et al. 2018). The different WT-boxes are characterized by their nucleotides directly adjacent to the GACTTT core sequence and may also contain the W-box TTGACC/T, the classic binding site of WRKY TFs (Rushton et al. 2010; Kanofsky et al. 2017). In some instance the WT-box is part of a *cis*-regulatory module (CRM) linked to W- and GCC-boxes (Lehmeyer et al. 2016; Kanofsky et al. 2017).

The binding of WRKY TFs to the WT-box was confirmed for different examples by using yeast one-hybrid screenings, transient reporter gene technology, and in vitro gel-shift assays. WRKY70 interacts, for example with the WT-box CGACTTTT (Machens et al. 2014) and also binds to the WT-box AGACTTTT in the SARD1 promoter (Zhou et al. 2017). Also, the WT-box GGACTTTC, present in a *cis*-regulatory module in the promoter of the *WRKY30* gene is bound by WRKY26, a transcriptional repressor of this CRM (Kanofsky et al. 2017). In another study which involved a CRM from the promoter of the *A. thaliana DJ1E* gene containing two WT-boxes and a GCC-box, yeast one-hybrid screenings only identified AP2/EREBP transcription factors interacting with the GCC-box (Lehmeyer et al. 2016). No factors binding to the WT-boxes GGACTTTT and GGACTTTG were identified in yeast one-hybrid screenings.

Recently, WRKY50 (At5g26170) was shown to interact with the promoter element GGACTTTTC in the *A. thaliana PR1* promoter (Hussain et al. 2018b). This promoter element harbours the WT-box core sequence GACTTT. However, WRKY50 was never identified in previous yeast one-hybrid screens with WT-box containing *cis*-sequences despite using a TF-only prey library (Mitsuda et al. 2010; Lehmeyer et al. 2016; Kanofsky et al. 2017). A recent yeast one-hybrid screen with a WT-box containing *cis*-sequence from the gene At1g76960 identified WRKY40, but this TF binds to the WT-box adjacent sequence TTTTCTA (Kanofsky et al. 2019).

WRKY50 is involved in the repression of jasmonic acid-mediated signalling under low oleic acid conditions (Gao et al. 2011). The WRKY50 encoding gene is a direct in vivo target of WRKY18, 33, and 40 (Birkenbihl et al. 2017). A model was proposed in which the phosphorylation status of WRKY50 associated with the activity of the *Arabidopsis* Botrytis-induced kinase I (BIKI) regulates jasmonic acid content (Lal et al. 2018). Overexpression of WRKY50 also leads to higher sinapic acid contents in transgenic plants (Hussain et al. 2018a). The target genes of WRKY50 were recently predicted by DAP-seq. DAP-seq identified genomic DNA fragments which can bind in vitro to WRKY50. Based on the frequency and genomic position of these sequences, a total of 10,181 putative WRKY50 target genes were proposed (O’Malley et al. 2016). The large number of WRKY50 target genes is surprising but is consistent with in vitro binding site and binding domain studies that showed and predicted a fairly high variability of WRKY50 binding sites (Brand et al. 2010, 2013). The DAP-seq study confirms *PR1* to be a target gene of WRKY50 (O’Malley et al. 2016). Interestingly, the proposed WRKY50 target genes *DJ1E* (At2g38860), *WRKY30* (At5g24110), and *ATBBE4* (At1g26390) all harbour WT-boxes in their promoters (Koschmann et al. 2012; Lehmeyer et al. 2016; O’Malley et al. 2016; Kanofsky et al. 2017)*.* This led to the hypothesis that these CRMs may be involved in WRKY50-regulated gene expression.

In the present study, the interaction of WRKY50 with WT-box containing CRMs from the WRKY50 target genes *DJ1E, WRKY30* and *ATBBE4* was investigated by transient reporter gene expression experiments and in vitro gel-shift assays. These analyses reveal that WT-boxes, whose sequences deviate from the classic W-box, are required for gene expression regulation and in vitro binding by WRKY50.

## Materials and methods

### Plasmid constructs

The recombinant reporter plasmids harbouring the tetramer of sequence 15 (S15) and S15mut1 through S15mut4 in pBT10GUS-d35SLUC were described previously (Lehmeyer et al. 2016). Also, tetramers of S20, S21, S22, S22mut1, S22mut2, S22mut3, S22mut4, S22mut5, S24, S24mut2, S24mut5, S24mut7, S24mut8, and S24mut9 in pBT10GUS-d35SLUC were described previously (Koschmann et al. 2012; Kanofsky et al. 2017). S15mut13 and S21mut1 through S21mut5 were synthesized as a monomer with partial *Spe*I and *Xba*I sites by Life Technologies (Darmstadt, Germany), annealed and ligated into the *Spe*I/*Xba*I sites of pBT10GUS-d35SLUC. Tetramerization was performed as described (Sprenger-Haussels and Weisshaar 2000; Rushton et al. 2002). The sequences of the monomers and of all mutations are shown in the figures without the *Spe*I/*Xba*I sites.

To construct the effector plasmid expressing WRKY50 (At5g26170), the cDNA for WRKY50 was generated from RNA isolated from *A. thaliana* Col-0 sprayed with 2 mM salicylic acid (Dong et al. 2003). Eight hours after spraying, RNA was isolated with the ‘NucleoSpin RNA Plant’ kit (Macherey-Nagel). The reverse transcriptase reaction was performed with the ‘RevertAid H Minus First Strand cDNA Synthesis Kit’ (Thermo Fisher Scientific, Rockford, IL, USA) on 1 µg RNA using oligo(dT) primers. The cDNA was amplified with WRKY50 specific forward and reverse primers 5′-CTTCCCGGGATGAATGATG-3′ and 5′-GTCGACTTAGTTCATGCTTGAGT-3′ using ‘peqGOLD Pwo-DNA-Polymerase’ (PEQLAB, Erlangen Germany) and cloned into pCR4Blunt-TOPO (‘Zero Blunt TOPO Cloning Kit for Sequencing’, Life Technologies). A *Sma*I/*Sal*I fragment harbouring the coding region from WRKY50 was inserted into the *Stu*I/*Sal*I site of plasmid pORE-O2-d35S-pA (pORE) (Machens et al. 2014). The recombinant plasmid was designated WRKY50-pORE.

For gel-shift assays WRKY50BD was expressed in *E. coli* BL21 using pQE-30 (Qiagen, Hilden, Germany). To amplify the DNA-binding domain (BD) from WRKY50-pORE primers 5′-CGCGGATCCCTGCCGACAACCAAAACAAG-3′ and 5′-CGCGTCGACTTAGTTCATGCTTGAGTGATTGTG-3′ were used. The amplified fragment was cut with *Bam*HI and *Sal*I and cloned into the *Bam*HI and *Sal*I sites from pQE-30. The resulting plasmid was designated WRKY50BD-pQE30.

Plasmid DNA for protoplast transformation was isolated as described by the manufacturer with the 'NucleoBond^R^ xtra midi EF' or 'NucleoBond^R^ xtra maxi kit' (Macherey-Nagel, Düren, Germany). For all recombinant DNA work, standard protocols were employed (Sambrook and Russell 2001). All cloning products were sequenced by GATC Biotech (Konstanz, Germany). DNA sequences were processed and analysed using the ‘CLC Main Workbench’ software (CLC Bio, Aarhus, Denmark).

### Transient reporter gene expression

For transient reporter gene expression analysis a parsley (*Petroselinum crispum*) cell culture was used and protoplasts were freshly prepared for each transformation experiment according to a published protocol (Kanofsky et al. 2016). For co-transformation experiments a TF-expressing effector construct based on pORE-O2-d35S-pA and reporter gene constructs harbouring the *cis*-regulatory sequences cloned as tetramers upstream of the minimal promoter of the *uid*A gene in pBT10GUS-d35SLUC (pBT10) were performed as described in Machens et al. (2014) and Lehmeyer et al. (2016). MAMP-responsive reporter gene assays were performed using recombinant pBT10GUS-d35SLUC reporter gene constructs with or without co-transformation of a TF-expressing effector plasmid into freshly prepared parsley protoplasts. Subsequently the transformed protoplasts were treated with or without the MAMP Pep25. As a positive control, the D-element cloned as a tetramer into pBT10GUS-d35SLUC was used (Koschmann et al. 2012). Quantification and normalization of reporter gene expression was done according to a recently published protocol (Kanofsky et al. 2016). All transformations were done at least three times independently with two technical replicates each. The exact number of experiments from which mean values and standard deviations were derived are given in the figure legends. Statistical differences between experiments were determined with a *t*-test. An asterisk in the figures denotes a significance threshold of ≤ 0.05 between two experimental conditions.

### Electrophoretic mobility shift assays

Recombinant WRKY50BD was expressed in and purified from *E. coli* BL21 (pREP4) using WRKY50BD-pQE30 as described earlier for WRKY70 and WRKY40 (Machens et al. 2014; Kanofsky et al. 2019). Oligonucleotides were end-labelled with P^32^ and used as probes in EMSAs (Hartmann Analytic, Braunschweig, Germany). The expression of WRKY50BD in *E. coli* was induced by adding IPTG to a final concentration of 1 mM at an optical density of 0.6 at 600 nm. After an incubation for 4 h the culture was precipitated by centrifugation. The bacteria were resuspended in 4 mL NPI-10 (50 mM NaH_2_PO_4_, 300 mM NaCl, 10 mM imidazole, pH 8) per gram cell pellet and Benzoase Nuclease (3 Units/mL, Sigma-Aldrich, München Germany), lysozyme (1 mg/mL), and ‘Halt Protease Inhibitor Cocktail, EDTA free (100 ×)’ (1 × final concentration, Thermo Fisher Scientific) were added (Machens et al. 2014; Kanofsky et al. 2018). The bacterial suspension was incubated on ice for 30 min. Subsequently, the volume was increased to 15 mL with NPI-10 and the bacteria were lysed with a French Pressure Cell Press. To purify recombinant WRKY50BD, the ‘Chelating Sepharose Fast Flow’ agent (GE Healthcare, München, Germany) was used. 1 mL Ni ions coated Sepharose in NPI-10 and the lysed bacterial cells were incubated for 1 h at 4 °C. The protein bound Sepharose was precipitated by centrifugation (500 *g*) and the pellet was washed with NPI buffer containing increasing concentrations of imidazole (10 mM, 20 mM, and 30 mM) followed by centrifugation and resuspension. In a last step the protein was eluted with 1 mL NPI-500 (500 mM imidazole) twice. The different fractions were analysed by SDS-Page, Coomassie staining and western blot. Protein concentration was determined using Bradford reagent. 4–5 µg of recombinant protein was used in a protein–DNA binding reaction. As binding reaction controls, also a sample with no protein or with *E. coli* protein from a strain containing only the vector was used. Gel electrophoresis, drying of the gel, and exposure to an X-ray film was done as described (Kanofsky et al. 2018). All EMSAs were performed three times. One exemplary result is shown in the figures.

## Results

### WRKY50 interacts with WT-box containing *cis*-regulatory sequences from WRKY50 target genes

**Fig. 1:**
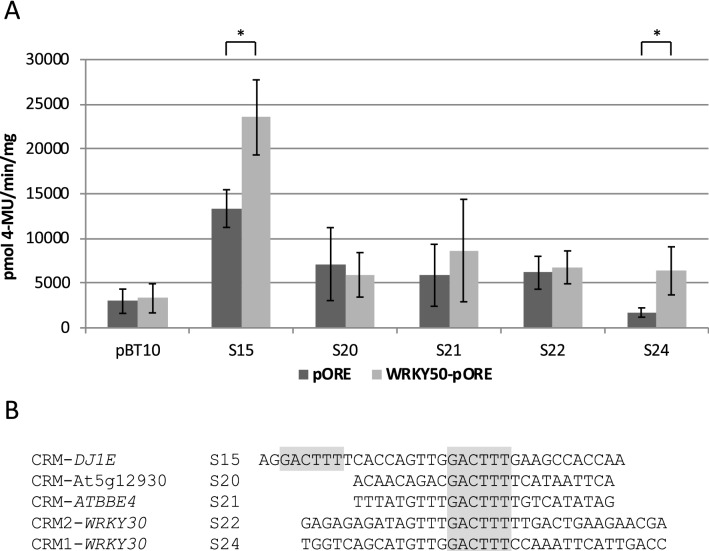
WRKY50 activates reporter gene expression through WT-box containing CRMs. **a** Transient reporter gene assays in parsley protoplasts after co-transformation of an effector plasmid expressing WRKY50 (WRKY50-pORE) or the empty vector (pORE) with reporter plasmids harbouring four copies of the indicated sequences upstream of the *uid*A (GUS) reporter gene. Relative GUS expression and standard deviations were determined from three (S15, S20, S21, S22, S24), or six (pBT10) independent experiments with technical duplicates, respectively. Sequences S20, S21, and S22 show no statistic significant difference of WRKY50 activation compared to the empty vector (pORE) activation, while S15, and S24 show a statistically significant WRKY50 activation (*p* < 0.001;*). **b** Sequences of the WT-box containing CRMs. The WT-box core sequences are marked in grey

The genes *DJ1E*, *ATBBE4*, and *WRKY30* are proposed target genes of WRKY50 and harbour WT-boxes similar to a WRKY50 binding site in their MAMP-responsive CRMs (Koschmann et al. 2012; Lehmeyer et al. 2016; O’Malley et al. 2016; Kanofsky et al. 2017; Hussain et al. 2018b). Since the identification of *DJ1E*, *WRKY30*, and *ATBBE4* as target genes for WRKY50 by DAP-seq analysis does not necessarily mean that the WT-box containing CRMs are within the fragments bound by WRKY50, we used the genome browser at the Cistrome database to determine their position. The obtained results confirmed that the WT-box containing CRMs from the proposed target genes *DJ1E*, *ATBBE4*, and *WRKY30* are all present within WRKY50-bound DNA fragments (O’Malley et al. 2016). Based on this observation, we asked the question whether WRKY50 can activate reporter gene expression through these CRMs. For this, transient reporter gene expression assays were performed employing a parsley protoplast system. Parsley protoplasts have been used as a well-established, standard system for transient gene expression analysis in all our previous work. Co-transformation experiments were done with a WRKY50 expressing effector plasmid (WRKY50-pORE) and reporter plasmids harbouring four copies of the CRMs upstream of the *uid*A (GUS) reporter gene in pBT10GUS-d35SLUC. As a control, a co-transformation with the empty effector plasmid (pORE) and a reporter plasmid void of *cis*-regulatory sequences (pBT10) was performed as well. Furthermore, a *cis*-regulatory sequence (S20) with a WT-box from a gene not predicted to be a WRKY50 target gene (At5g12930) was investigated as well. Following the transformation, GUS reporter gene activity was determined and normalized using a constitutively expressed luciferase gene on the reporter gene plasmid (Kanofsky et al. 2016). Figure 1 shows the result of the transient reporter gene assays (A) and the sequences of the analysed CRMs (B). CRM2-WRKY30 was investigated in the opposite orientation as displayed in Fig. 1. This is consistent with the orientation of CRM2-*WRKY30* in the *WRKY30* promoter (Kanofsky et al. 2017). The orientation displayed in Fig. 1 and below is chosen for better comparability. As expected, in transient reporter gene assays neither the empty effector plasmid pORE, nor the effector plasmid expressing WRKY50 has an effect on the empty reporter plasmid pBT10 and *cis*-sequence S20. In the presence of CRM-*DJ1E* (S15) and CRM1-*WRKY30* (S24), however, the effector plasmid expressing WRKY50 enhances reporter gene activity (Fig. 1). CRM2-*WRKY30* (S22) and CRM-*ATBBE4* (S21) do not show significantly altered reporter gene expression in the presence of WRKY50 (Fig. 1). In summary, these data suggest that WT-boxes represent potential targets of WRKY50 mediating gene activation.

### WRKY50-activated reporter gene expression requires the WT-boxes GGACTTTT, GGACTTTG and GGACTTTC

**Fig. 2:**
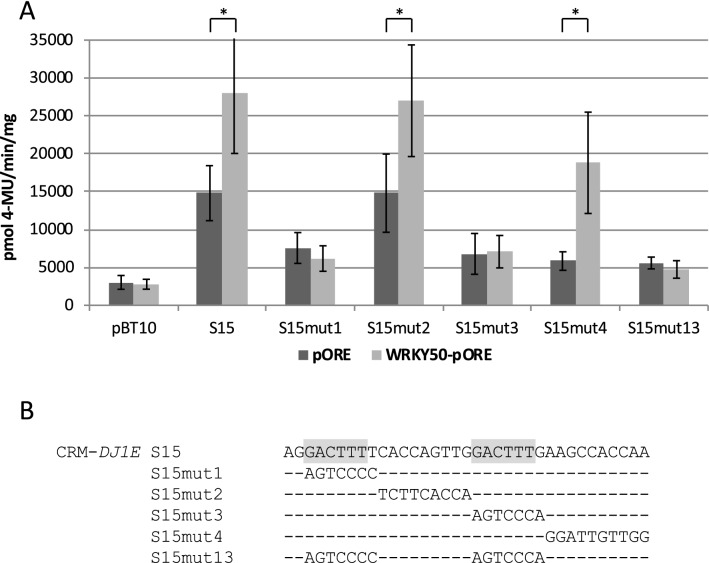
WRKY50 requires the WT-boxes in CRM-*DJ1E* for reporter gene activation. **a** Transient reporter gene assays in parsley protoplasts as described in Fig. 1. Relative GUS expression and standard deviations were determined from three (pBT10, S15mut1, S15mut4, S15mut13), four (S15mut2, S15mut3), or ten (S15) independent experiments with technical duplicates, respectively. Mutations S15mut1, S15mut3, and S15mut13 show no significant statistical differences of WRKY50 activation compared to the empty vector (pORE) activation, while S15 and mutations S15mut2 and S15mut4 show a statistically significant WRKY50 activation (*p* < 0.001;*). **b** Sequence of S15 and of five mutations. The WT-box core sequences are marked in grey. Altered nucleotides in the sequence are shown, unaltered nucleotides are not shown (−)

As shown in Fig. 1, WRKY50 can activate reporter gene expression through CRM-*DJ1E* (S15) and CRM1-*WRKY30* (S24). To determine if the WT-boxes in these CRMs are required for WRKY50-responsive reporter gene expression, mutations in CRM-*DJ1E* and CRM1-*WRKY30* were analysed for their effect on gene upregulation by WRKY50. Figure 2 shows that a single mutation in either of the two WT-boxes and a double mutation in both WT-boxes of the CRM-*DJ1E* abolishes WRKY50-responsive reporter gene expression (S15mut1, S15mut3, and S15mut13). In contrast, a mutation between both WT-boxes and a mutation downstream of the WT-box have no effect on WRKY50-stimulated reporter gene expression (S15mut2 and S15mut4). Taken together, these observations indicate, that both WT-boxes are required for WRKY50-activated reporter gene expression. A single WT-box is therefore not sufficient for WRKY50-mediated gene activation.

**Fig. 3:**
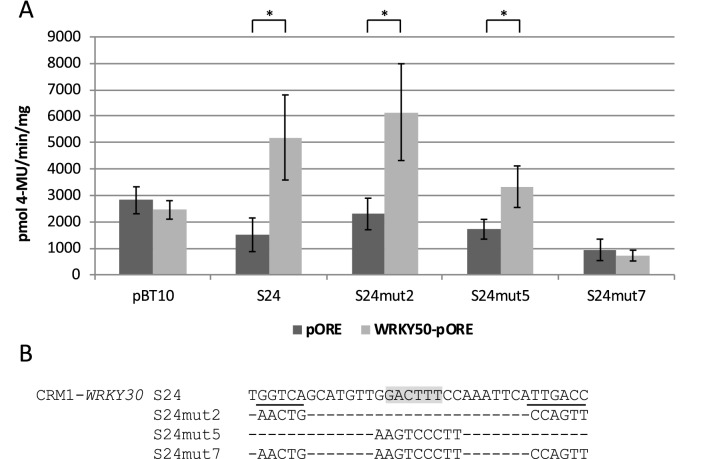
WRKY50 requires the WT-box in CRM1-*WRKY30* for reporter gene activation. **a** Transient reporter gene assays in parsley protoplasts as described in Fig. 1. Relative GUS expression and standard deviations were determined from three (pBT10, S24mut2, S24mut5, S24mut7), or six (S24) independent experiments with technical duplicates, respectively. Mutation S24mut7 shows no significant statistical difference of WRKY50 activation compared to the empty vector (pORE) activation, while S24 and mutations S24mut2 and S24mut5 show a statistically significant WRKY50 activation (*p* < 0.001;*). **b** Sequence of S24 and of three mutations. The WT-box core sequence is marked in grey and the W-boxes are underlined. Altered nucleotides in the sequence are shown, unaltered nucleotides are not shown (−)

To further test the hypothesis that the WT-box mediates gene activation through WRKY50, CRM1 of *WRKY30* were also tested by targeted mutation. CRM1-*WRKY30* harbours one WT-box and two W-box or W-box like sequences. One W-box is complete (TTGACC) while a partial one is located in the opposite orientation and lacks the T at its 5′ end (TGACC). Figure 3 shows that mutations in both W-boxes (S24mut2) do not impact WRKY50-responsive reporter gene activation compared to the unmutated sequence (S24). This suggests that the WT-box in S24 is sufficient for reporter gene activation in response to WRKY50. Consistent with this notion, a mutation in the WT-box (S24mut5) strongly reduces reporter gene induction. Interestingly, however, gene activity is not fully abolished in this construct. Only mutations in both W-boxes and the WT-box (S24mut7) fully suppress WRKY50-mediated reporter gene activation. This indicates that both W-boxes also contribute to WRKY50-activated reporter gene expression. Mutations in both W-boxes and the WT-box (S24mut7) abolish WRKY50-mediated reporter gene activation.

In summary, the WT-boxes GGACTTTT and GGACTTTG in CRM-*DJ1E* and the WT-box GGACTTTC in CRM1-*WRKY30* are required for WRKY50-activated reporter gene expression.

### WRKY50 affects MAMP-responsive reporter gene expression by CRM2-*WRKY30*

**Fig. 4:**
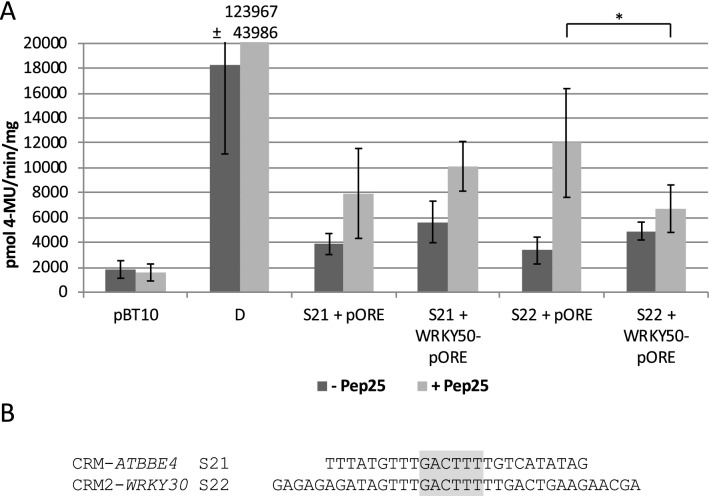
WRKY50 affects MAMP-responsive reporter gene activation by CRM2-*WRKY30.* **a** Transient reporter gene assays in parsley protoplasts after co-transformation of plasmids harbouring CRM-*ATBBE4* (S21) and CRM2-*WRKY30* (S22) with a WRKY50 expressing (WRKY50-pORE) or a non-expressing plasmid (pORE) in the presence and absence of Pep25. The empty vector pBT10GUS-d35SLUC (pBT10) and this vector containing four copies of the D-element (D) were used as a negative and positive control for Pep25 induction, respectively. Relative GUS expression and standard deviations were obtained from three (S21), four (S22), or seven (pBT10, D) independent experiments with technical duplicates, respectively. Statistically significant reduction of Pep25 induction in the presence of WRKY50 is indicated with S22 (*p* < 0.05;*). **b** Sequence of S21 and S22

As shown in Fig. 1, WRKY50 does not activate reporter gene expression through CRM-*ATBBE4 *(S21) and CRM2-*WRKY30* (S22). Therefore, it was investigated if co-expression of WRKY50 in the presence and absence of the MAMP Pep25 in parsley protoplasts affects MAMP-responsive reporter gene expression. For this, transient reporter gene expression assays after co-transformation of the WRKY50 expressing effector plasmid (WRKY50-pORE) and reporter plasmids harbouring four copies of CRM-*ATBBE4 *(S21) and CRM2-*WRKY30* (S22) upstream of the *uid*A (GUS) reporter gene were performed. Pep25 is an oligopeptide derived from a surface glycoprotein of the phytopathogenic oomycete *Phytophthora sojae* (Nürnberger et al. 1994; Rushton et al. 1996). As a control, co-transformation with the empty effector plasmid (pORE) and a reporter plasmid void of *cis*-regulatory sequences (pBT10) or harbouring four copies of the Pep25-responsive D-element were performed as well. The D-element was identified in the parsley *PR2* gene promoter (van de Löcht et al. 1990; Kirsch et al. 2000; Rushton et al. 2002). As shown in Fig. 4, the empty vector (pBT10) does not show any Pep25-responsive gene expression while the D-element (D) shows strong Pep25 responsivity. In case of CRM-*ATBBE4 *(S21) and CRM2-*WRKY30* (S22) Pep25-responsive gene expression is observed. In the presence of WRKY50, only Pep25-responsive reporter gene activation of CRM2-*WRKY30* but not CRM-*ATBBE4* is negatively affected*.* This may indicate a negative effect of WRKY50 on the MAMP responsivity of CRM2-*WRKY30* but does not proof the physical interaction of WRKY50 with either CRM-*ATBBE4 *or CRM2-*WRKY30*.

### The WT-boxes in all four CRMs are direct binding sites of WRKY50

**Fig. 5:**
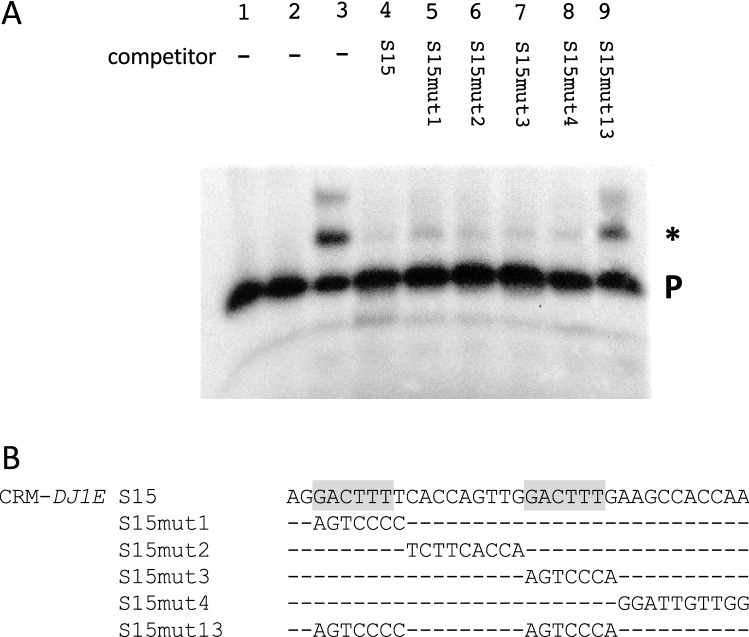
Binding of WRKY50BD to CRM-*DJ1E* requires the WT-boxes GGACTTTT and GGACTTTG. **a** Electrophoretic mobility shift experiment with WRKY50 and the CRM-*DJ1E* (S15) as probe. Lane 1: free probe. Lane 2: free probe plus purified protein extract of *E. coli* not expressing WRKY50BD. Lane 3: free probe plus purified WRKY50BD. Lane 4: free probe, purified WRKY50BD, and unlabelled competitor (S15) in a 100-fold molar excess. Lanes 5–9: free probe, purified WRKY50BD, and unlabelled mutations S15mut1, S15mut2, S15mut3, S15mut4, and S15mut13, respectively, in a 100-fold molar excess. A P designates the position of the free probe and a specific DNA–protein complex is marked by an asterisk (*). **b** The sequence of S15 and of five mutations are shown as in Fig. 2

**Fig. 6:**
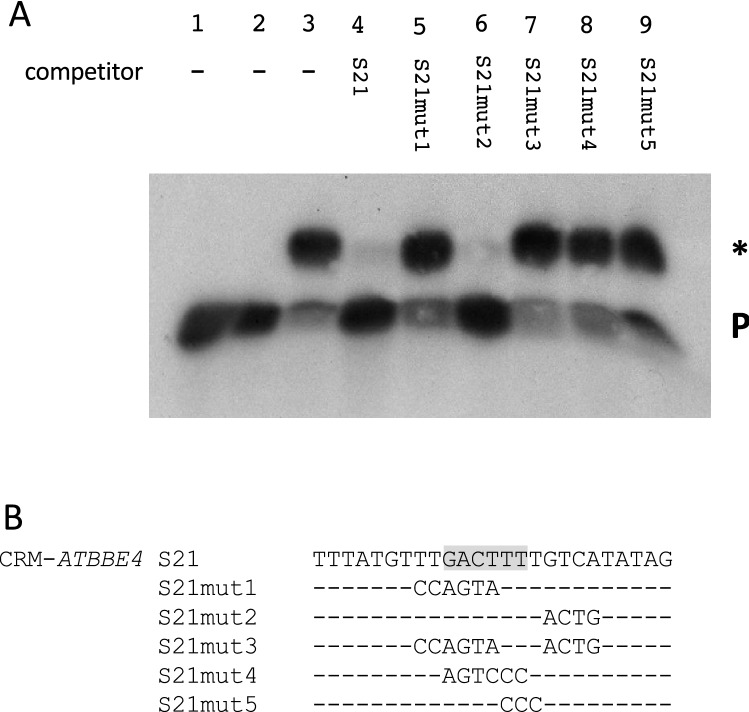
Binding of WRKY50BD to CRM-*ATBBE4* requires the WT-box TTGACTTTT. **a** Electrophoretic mobility shift experiment with WRKY50BD and the CRM-*ATBBE4* (S21) as probe. Lanes 1–4 are as in Fig. 5 except the labelled probe and the unlabelled competitor (lane 4) is S21. Lanes 5–9: free probe, purified WRKY50BD, and unlabelled mutations S21mut1, S21mut2, S21mut3, S21mut4, and S21mut5, respectively, in a 100-fold molar excess. A P designates the position of the free probe and a specific DNA–protein complex is marked by an asterisk (*). **b** The sequence of S21 and of five mutations are shown. The WT-box core sequence is marked in grey. Altered nucleotides in the sequence are shown, unaltered nucleotides are not shown (−)

**Fig. 7:**
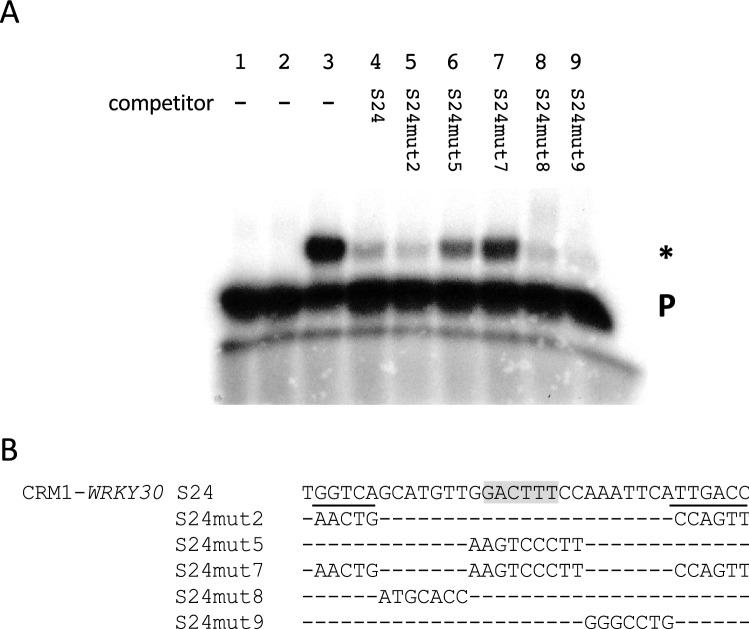
Binding of WRKY50BD to CRM1-*WRKY30* requires the WT-box GGACTTTC. **a** Electrophoretic mobility shift experiment with WRKY50BD and the CRM1-*WRKY30* (S24) as probe. Lanes 1–4 are as in Fig. 5 except the labelled probe and the unlabelled competitor (lane 4) is S24. Lanes 5–9: free probe, purified WRKY50BD, and unlabelled mutations S24mut2, S24mut5, S24mut7, S24mut8, and S24mut9, respectively, in a 100-fold molar excess. A P designates the position of the free probe and a specific DNA–protein complex is marked by an asterisk (*). **b** The sequences of S24 and of five mutations are shown. The WT-box core sequence is marked in grey and the two W-boxes are underlined. Altered nucleotides in the sequence are shown, unaltered nucleotides are not shown (−)

**Fig. 8:**
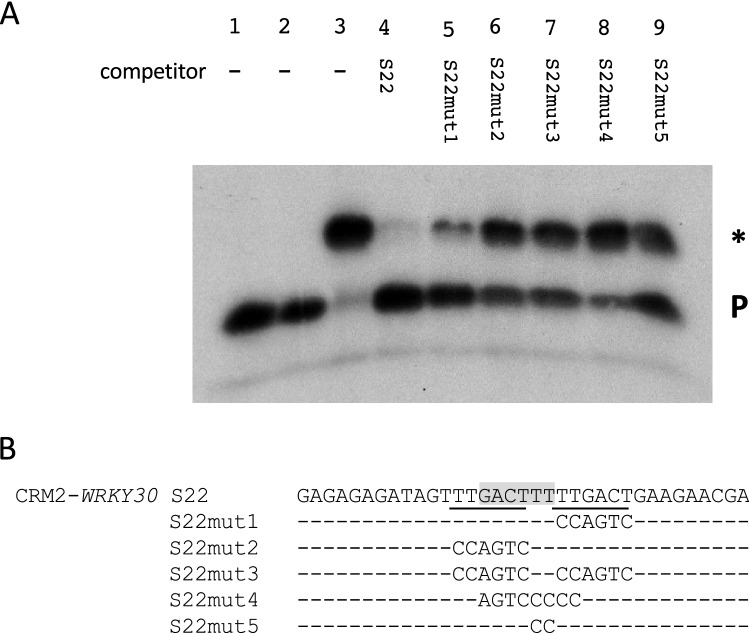
Binding of WRKY50BD to CRM2-*WRKY30* requires the WT-box TTGACTTTT. **a** Electrophoretic mobility shift experiment with WRKY50BD and the CRM2-*WRKY30* (S22) as probe. Lanes 1–4 are as in Fig. 5 except the labelled probe and the unlabelled competitor (lane 4) is S22. Lanes 5–9: free probe, purified WRKY50BD, and unlabelled mutations S22mut1, S22mut2, S22mut3, S22mut4, and S22mut5, respectively, in a 100-fold molar excess. A P designates the position of the free probe and a specific DNA–protein complex is marked by an asterisk (*). **b** The sequences of S22 and of five mutations are shown. The WT-box core sequence is marked in grey and the W-boxes are underlined. Altered nucleotides in the sequence are shown, unaltered nucleotides are not shown (−)

The regulatory sequences CRM-*DJ1E* (S15) and CRM2-*WRKY30* (S24) activate reporter gene expression by WRKY50 in transient expression studies (Fig. 1). In contrast, WRKY50 does not activate CRM2-*WRKY30* (S22) and CRM-*ATBBE4* (S21)-mediated reporter gene expression (Fig. 1). To determine if these four CRMs are directly bound to WRKY50 in vitro and if this binding is WT-box specific, in vitro gel-shift or EMSA experiments were performed with radioactively labelled sequences subjected to specific competition experiments by an excess of mutated or unmutated sequences. Figures 5, 6, 7, 8 show the results of these experiments for CRM-*DJ1E* (S15), CRM-*ATBBE4* (S21), CRM1-*WRKY30* (S24), and CRM2-*WRKY30* (S22), respectively. In these experiments the CRMs were radioactively labelled and subjected to binding studies using the DNA-binding domain of WRKY50 (WRKY50BD) purified from *E. coli* (Materials and methods). As reported before, the full length WRKY50 could not be used for in vitro binding studies (Hussain et al. 2018b). This became evident in EMSA experiments using the 88-bp C-terminal DNA-binding domain and the full length WRKY50 on an 80 bp *PR1* promoter fragment. Only the C-terminal DNA-binding domain produced a shift (Hussain et al. 2018b). In a similar experiment using the *cis*-sequence CRM-*ATBBE4 *(S21), no shift was observed with the full length WRKY50 but only with the WRKY50BD (data not shown). Therefore, WRKY50BD was used for all EMSA experiments in our study.

In case of CRM-*DJ1E* (S15) a shifted complex is observed in the presence of WRKY50BD (Fig. 5, lane 3). This signal can be abolished with an excess of unlabelled S15 (lane 4). Mutations in which one or both WT-boxes are left intact (S15mut1, 2, 3, and 4) can still abolish the shift indicating that one WT-box is sufficient for WRKY50BD binding. If both WT-boxes are mutated (S15mut13), the shift cannot be abolished, indicating that WRKY50BD binds to both WT-boxes (Fig. 5). While for in vitro binding a single WT-box seems to be sufficient, a single WT-box is not sufficient for activating reporter gene expression by WRKY50.

As shown in Fig. 1, CRM-*ATBBE4* (S21) does not have an effect on WRKY50-mediated reporter gene activation although CRM-*ATBBE4* is derived from a WRKY50 target gene. In contrast to the transient expression analysis, WRKY50BD binds directly to CRM-*ATBBE4* (S21) in vitro. Figure 6, lane 3 shows a shifted complex in the presence of WRKY50BD, which can be competed with an excess of unlabelled S21 (lane 4). Mutations affecting the WT-box can no longer abolish complex formation (S21mut1, 3, 4, and 5) while a mutation downstream of the WT-box (S21mut2) can still abolish complex formation. This shows that WRKY50BD directly binds to the WT-box in S21 in vitro, although no effect on transient reporter gene expression is observed in the presence of WRKY50.

CRM1-*WRKY30* (S24) and CRM2-*WRKY30* (S22) both affect reporter gene expression in the presence of WRKY50 (Figs. 1, 3, 4). Figure 7 shows WRKY50BD binding to S24 in vitro. Lane 3 shows a shifted complex in the presence of WRKY50BD which can be abolished with an excess of unlabelled S24 (lane 4). Competition with mutated fragments in which the WT-box is intact (S24mut2, 8, and 9) can abolish the shift indicating that the WT-box is sufficient for WRKY50BD binding. With competitors containing a mutated WT-box (S24mut5 and 7), a shift can be observed indicating that sequences with a mutated WT-box no longer bind to WRKY50BD efficiently. The difference in competition efficiency between S24mut5 and S24mut7 may indicate that a sequence in which both W-boxes are intact (S24mut5) can still partially compete with WRKY50 binding, while a sequence in which all three boxes are mutated (S24mut7) no longer abolishes WRKY50BD binding (Fig. 7).

To investigate WRKY50BD binding to CRM2-*WRKY30* (S22) and to determine the binding site specificity of WRKY50 within S22, similar EMSA experiments were performed with S22. Figure 8 shows binding of WRKY50BD to S22 (lane 3) and that binding can be abolished with an excess of unlabelled competitor (lane 4). Mutations affecting the WT-box (S22mut1-5) can no longer abolish the shift (Fig. 8, lanes 5–9). S22 harbours two complete W-boxes (TTGACT) of which one is part of the WT-box and the other one is adjacent to the WT-box. When a mutation within the adjacent W-box (S22mut1) is used as a competitor, the competition is less efficient indicating a contribution of the W-box in WRKY50 binding (lane 5). However, a mutation leaving the W-box intact, is unable to abolish the shift (S22mut2, lane 6). These results indicate the essential role of the WT-box TGACTTTT for WRKY50BD binding (Fig. 8). In all four cases, CRM-*DJ1E* (S15), CRM-*ATBBE4* (S21), CRM1-*WRKY30* (S24), and CRM2-*WRKY30* (S22), WRKY50BD directly interacts with the WT-boxes GGACTTTT, GGACTTTG, GGACTTTC and TGACTTTT, respectively.

## Discussion

### WRKY50 interacts with a CRM from the *DJ1E* gene to activate reporter gene expression

The CRM-*DJ1E* was originally identified as a 35 bp long *cis*-sequence (S15) showing strong MAMP-responsive reporter gene activity in parsley protoplasts (Koschmann et al. 2012). The *cis*-sequence is located in the upstream region of two gene models of the *DJ1E* gene (At2g38860.1 and At2g38860.3) while also located in the upstream untranslated region of At2g38860.2 (Lamesch et al. 2012). *DJ1E* encodes the DJ1 protein homologue E which is a member of the *DJ1* gene family (Lin et al. 2011). The *DJ1* gene family of *A. thaliana* contains homologues of the human oncogene *DJ1* (Nagakubo et al. 1997). Mutations in DJ1 are responsible for the onset of familial Parkinson’s disease and DJ1 is involved in antioxidant stress to prevent cell death (Bonifati et al. 2003; Taira et al. 2004). Not much is known about the function of the six *A. thaliana DJ1* family members (Seo et al. 2012). The proteins DJ1A, B and D have glyoxalase activity in vitro*,* but no glyoxalase activity was found for DJ1C, E, and F (Kwon et al. 2013). Similar to its human homologue, *DJ1A* protects against oxidative stress by activation of cytosolic superoxide dismutase (Xu et al. 2010). *DJ1C* is essential for chloroplast development (Lin et al. 2011) and *DJ1E,* also designated yellow-leaf-specific gene *5* (*YLS5*), is upregulated in late senescence stages (Yoshida et al. 2001). A role for *DJ1E* in camalexin and tryptophan biosynthesis pathways was suggested because *DJ1E* is co-expressed with tryptophan biosynthesis pathway genes and with phytoalexin deficient 3 (*PAD3*) (Wei et al. 2006). Recently, *DJ1E* was among a set of genes shown to be responsive to cyclic AMP concentrations (Sabetta et al. 2019). Although the *DJ1E* gene is upregulated by a diverse set of biotrophs and necrotrophs (Zimmermann et al. 2004; Bülow et al. 2007), the mechanism for *DJ1E* upregulation has not been elucidated. The analysis of the *cis*-regulatory module CRM-*DJ1E* revealed the presence of three *cis*-regulatory sequences, two WT-boxes and one GCC-box that are all required for MAMP-induced reporter gene expression (Lehmeyer et al. 2016). Yeast one-hybrid screens identified two AP2/EREBP TFs that play antagonistic roles as activator and repressor of CRM-*DJ1E*-regulated reporter gene expression through the GCC-box. However, no TF interacting with the WT-boxes had been identified so far (Lehmeyer et al. 2016). The recent analysis of the *PR-1* promoter of *A. thaliana* revealed a putative candidate TF for interaction with the WT-boxes of the CRM-*DJ1E* (Hussain et al. 2018b). WRKY50 was shown to interact with the sequence GGACTTTTC in the *PR-1* promoter, which was also shown to be involved in 2,6-dichloroisonicotinic acid (INA)-regulated gene expression (Lebel et al. 1998). This sequence is very similar to the WT-boxes in the CRM-*DJ1E* (Fig. 2). These findings prompted us to investigate the interaction of WRKY50 with the CRM-*DJ1E.* The obtained data revealed that both WT-boxes are required for WRKY50-activated reporter gene expression (Fig. 2) and for direct binding in gel-shift assays (Fig. 5). The binding of WRKY50 to the WT-boxes GGACTTTT and GGACTTTG seemed to be surprising since the WT-box deviates from the classic WRKY binding site TTGACC/T. However, previous in vitro binding site and binding domain studies showed and proposed a fairly high variability of WRKY50 binding site recognition (Brand et al. 2010, 2013). These earlier studies suggested that WRKY50 seems to require only a conserved GAC core sequence to interact with DNA (Brand et al. 2013). This hypothesis is not supported by our data since the transitions used for generating mutations also generates a GAC core sequence on the opposite strand. For example, the sequence GACTTT in CRM-*DJ1E* was mutated to yield AGTCCC which maintains a minimal GACT core sequence of a W-box on the opposite strand. Since these mutations abolish WRKY50-activated gene expression, the adjacent nucleotides and/or the orientation of the sequence are important (Fig. 2). Nevertheless, the high variability in binding site recognition is consistent with the large number of 10,181 putative WRKY50 target genes determined by DAP-seq (O’Malley et al. 2016). Among these are the target genes *DJ1E*, *ATBBE4*, and *WRKY30* that are shown here to harbour WRKY50 target sites within CRMs in their upstream regulatory regions.

### Interaction of WRKY50 with the CRM-*ATBBE4*

The CRM-*ATBBE4* was originally identified as S21 in the course of isolating novel MAMP-responsive *cis*-regulatory sequences (Koschmann et al. 2012). ATBBE4 belongs to a family of berberine bridge enzyme-like (BBE-like) proteins present in plants, fungi, and bacteria (Daniel et al. 2016). The berberine bridge enzyme-like gene family harbours 28 genes of which two catalyse the oxidation of aromatic allylic alcohols to the corresponding aldehydes (Daniel et al. 2015).

Recently, a BBE-like protein was shown to oxidize cellulose oligomers and to play a role in plant immunity (Locci et al. 2019). *ATBBE4* encodes a cytoplasmic and extracellular FAD-binding protein with oxidoreductase activity. *ATBBE4* is upregulated upon infection by bacterial, fungal, and oomycete pathogens and downregulated in response to abiotic stimuli (Bülow et al. 2007; Hehl et al. 2013). Since the CRM-*ATBBE4* confers MAMP-responsive reporter gene expression (Koschmann et al. 2012), this CRM may be responsible for the upregulation of gene expression by biotic stimuli. It is tempting to speculate upon the role of WRKY50 in regulating *ATBBE4* gene expression because *ATBBE4* is a proposed target gene of WRKY50 (O’Malley et al. 2016) and the CRM-*ATBBE4* is bound by WRKY50 in vitro (Fig. 6). However, transient reporter gene expression studies neither show activation nor repression of reporter gene expression in the presence of WRKY50 (Figs. 1, 4). This may be attributed to a low sensitivity of the transient reporter gene expression system. Therefore, it may be interesting to study the effect of WRKY50 on *ATBBE4* gene expression in transgenic plants overexpressing or lacking WRKY50.

### WRKY30, a target for WRKY50-regulated gene expression

The two MAMP-responsive CRMs from the *WRKY30* promoter were previously analysed in detail (Kanofsky et al. 2017). Using yeast one-hybrid screenings, WRKY26, WRKY40, WRKY41, and WRKY70 were identified with CRM1-*WRKY30*. While WRKY70 is a transcriptional activator of CRM1-*WRKY30*, WRKY26, WRKY40, and WRKY41 downregulate MAMP-responsive reporter gene expression by CRM1-*WRKY30* (Kanofsky et al. 2017). Later it was shown by in vitro gel-shift assays that direct binding of WRKY26 to CRM1-*WRKY30* requires the WT-box and both W-boxes (Kanofsky et al. 2018). Based on the results here it is surprising that WRKY50 was not selected in yeast one-hybrid screenings with the CRM1-*WRKY30*. This may be explained by the observation that a full length WRKY50 did not interact with DNA. Based on homology modelling for both full length and truncated proteins, the absence of an interaction with the full length protein may be due to the N-terminal region which possibly impedes the binding of DNA (Hussain et al. 2018b). Because WRKY50 was shown to be regulated by phosphorylation, this may affect its DNA-binding property (Lal et al. 2018).

WRKY30 is a member of the group III family of WRKY TFs. Group III members can be distinguished from members of group I and II by their distinct zinc finger motif (Eulgem et al. 2000). WRKY30 is induced within 2–6 h after inoculation with two *Peronospora parasitica* isolates and also induced by *Blumeria graminis* f. sp. *hordei* (Kalde et al. 2003). Upregulation by these pathogens is not induced by salicylic acid (SA) and independent from SA signalling although it has been found that WRKY30 upregulation during senescence may be partially SA dependent (Besseau et al. 2012). Although upregulation is observed by *P. parasitica*, upregulation does not seem to depend on signalling by the *P. parasitica* resistance genes RPP2 and RPP4 (Kalde et al. 2003). These conclusions are based on the analysis of mutants defective in SA and RPP2/RPP4 signalling. In contrast, WRKY30 expression depends on chitin signalling. In a chitin receptor kinase mutant (*cerk1*), WRKY30 is no longer induced indicating WRKY30 is upregulated upon chitin perception (Cao et al. 2014). When WRKY30 induction is analysed for biotic and abiotic stimuli using the database PathoPlant, strong induction of WRKY30 is observed during salt-stress (Bülow et al. 2007). The involvement of WRKY30 in abiotic stress pathways was further substantiated by its overexpression in *A. thaliana* and wheat (*Triticum aestivum*). During seed germination in *A. thaliana* plants overexpressing WRKY30 were more tolerant to oxidative and salinity stress than wild-type plants (Scarpeci et al. 2013). In wheat, overexpression of WRKY30 leads to enhanced heat and drought stress tolerance (El-Esawi et al. 2019). Enhanced heat stress tolerance is conceivable in the light of WRKY30 transcriptional induction by the heat shock factor A4A which itself is a target for mitogen-activated protein kinase 4 phosphorylation (Perez-Salamo et al. 2014; Andrasi et al. 2019). WRKY50 may be a second TF regulating WRKY30 gene expression during abiotic stress.

#### Author contribution statement

RH conceived the idea. KK, JR, LE, FM performed the experiments and analysed the data. RH and KK wrote the manuscript. All authors read and approved the final manuscript.
